# Supplementary material for: The Gut Bacteria Dysbiosis Contributes to Chronic Graft-Versus-Host Disease Associated With a Treg/Th1 Ratio Imbalance
Source: Front Microbiol. 2022 Sep 8;13:813576. doi: 10.3389/fmicb.2022.813576 (PMC9493085; doi:10.3389/fmicb.2022.813576)
Supplement: Supplementary file 1 [file Data_Sheet_1.zip › P101SC18090073-01-B1-3-4_result/04.BetaDiversity/NMDS/NMDS_with_cluster.pdf]

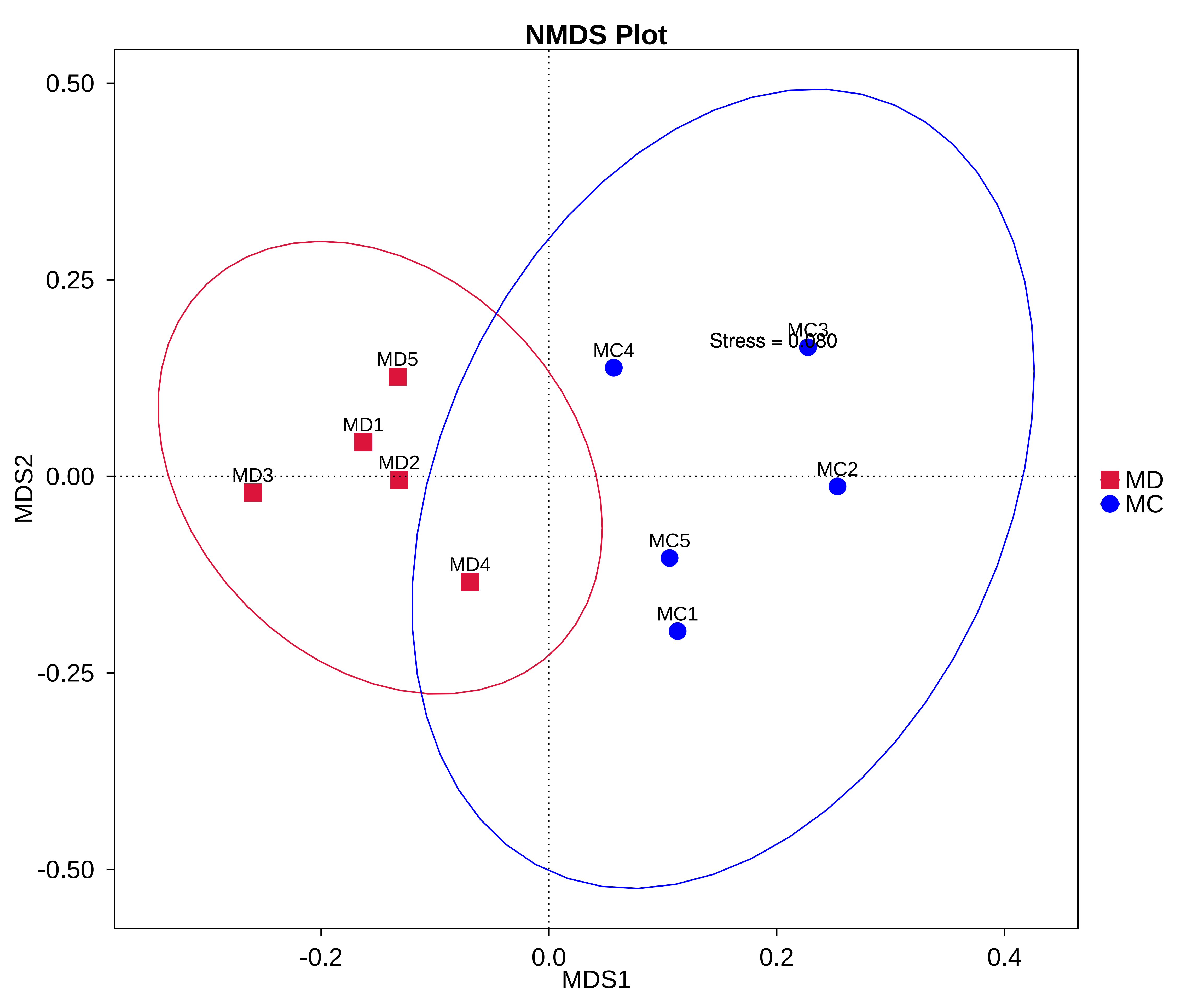
NMDS Plot
0.50
0.25
0.00
-0.25
-0.50
MDS2
-0.2
0.0
0.2
0.4
MDS1
MD5
MD1
MD2
MD3
MD4
MC4
MC3
Stress = 0.080
MC2
MC5
MC1
MD
MC
